# Readiness for Self-Directed learning among first year Saudi Medical students: A descriptive study

**DOI:** 10.12669/pjms.314.7057

**Published:** 2015

**Authors:** Mona Soliman, Ghadeer Al-Shaikh

**Affiliations:** 1 Dr. Mona Soliman, Department of Physiology, College of Medicine, King Saud University, Riyadh 11461, Saudi Arabia; 2 Dr. Ghadeer Al-Shaikh, Department of Obstetrics and Gynecology, College of Medicine, King Saud University, Riyadh 11461, Saudi Arabia College of Medicine, Princess NourahBintAbdulrahman University

**Keywords:** Self-directed learning, Medical Students

## Abstract

**Objectives:**

The objectives of the present study was to explore the readiness for Self Directed Learning (SDL) among first year Saudi Medical students enrolled at King Saud University (KSU) and Princess NourahBintAbdulrahman University (PNU) in Saudi Arabia.

**Methods:**

First year medical students were invited to participate in a descriptive cross sectional study design. Data were collected using a Self-Directed Learner Readiness Scale (SDLRS) which is a self- assessment tool aimed to assess three main components: self-management, desire for learning and self-control. The students responded to each item of the SDLRS on a 5-point Likert scale. Data were analyzed using SPSS, mean, median and total scores were calculated and were compared among student’s groups.

**Results:**

The mean score for the desire of learning was the highest (4.08± 0.5) of all the three components of the SDLRS followed by self-control (3.9± 0.9), while the least mean score was for self-management (3.7±0.5). Overall, differences between student’s groups were not statistically significant.

**Conclusion:**

The present study revealed that the overall SDL readiness of participants was good, students were highly motivated for self-learning and had the ability for self-control. However, they need assistance to improve their self-management skills.

## INTRODUCTION

Self-directed learning (SDL) is an important skill for medical students and it is considered as an integral part of student centered medical curricula.^1^-^4^ Shortened lifespan of relevant information and increased complexity of clinical practice make SDL virtually important for health professionals to retain competency by continuous learning and seeking for knowledge updates throughout their careers. Knowles defined SDL as ‘a process in which individuals take the initiative with or without the help of others in diagnosing their learning needs, formulating goals, identifying human and material sources, and evaluating learning outcomes’.^5^ SDL is defined as the process of deciding what to learn to what depth and breadth. The self-directed learners take control and accepts the freedom to learn what they view as important for themselves.^6^-^8^ The degree of control the learners are willing to take over their own learning will depend on their attitude, abilities and personality characteristics.

SDL is an embedded part of the innovative medical curricula that adopt the problem-based learning (PBL). PBL is viewed as an innovative strategy that changes the teaching context from teacher-centered learning to student-centered. In medical curricula the system is based on real cases that demonstrate core learning objectives with integration of basic and clinical sciences knowledge. Students enrolled in such system must have the competencies of SDL. The instrument most widely used to measure SDL readiness is Guglielmino’s self-directed learning readiness scale (SDLRS).

KSU and PNU Medical Colleges, are implementing the same innovative reformed curriculum that adapts PBL strategy, where SDL time constitutes an important part of the curriculum. Therefore assessing junior student’s SDL competencies are considered an essential part of the ongoing monitoring of the recently reformed program. It will also shed the light on areas of improvement concerning the main domains of SDL during early phases of the program where intervention could be possible. The present study was designed to explore the SDL readiness of the first year medical students in KSU and PNU.

## METHODS

### Study Design and study settings

This is a cross-sectional survey design using a convenient sample of first year medical students enrolled in KSU and PNU. Students from both KSU and PNU amounted to 400; 134 females and 194 males were from KSU and 69 females from PNU. The medical program is a five year curriculum with the first two years of basic sciences focusing on nine blocks, based on systems and divided into four semesters. The third year is a transition between the basic science and the two years of clinical clerkship. During the first two years, the courses share full vertical and horizontal integration of basic science subjects: Anatomy, Physiology, and Biochemistry with Pathology, Microbiology, Pharmacology and few relevant topics of Community Medicine according to the themes of weeks. Teaching strategies include interactive lectures, tutorials, practical and demonstration, smart lab, history taking and PBL sessions. The design of PBL is based on real cases that demonstrates core-learning objectives with integration of basic and clinical sciences knowledge. Times for SDL constitute an integral part of the curriculum and are considered on daily basis

Before the start of data collection, the study was submitted to Ethical Review Board at KSU and PNU for approval. During data collection, verbal permission was taken from all participants.

### Data Collection

SDLRD questionnaire was used to collect the data; the scale was first developed and tested by Fisher et al.^6^ It is self-perception scale in English language including 40-items grouped under three subscales and measuring three components; (1) 13 items for self-management, (2) 12 items for the desire for learning and (3) 15 items for self-control. Responses to 36 items were ordinal on a five-point Likert scale format, where 1 indicated strongly disagree and 5 indicated strongly agree, the other four items were scored in a reverse order (items 3, 11, 20 and 40).

Questionnaires were distributed by the researchers who gave detailed instructions on how to complete the questionnaire and informed the students about the objectives of the study. Students were also assured on confidentiality of the questionnaire and that no harm or legal consequences will issue regarding the results of the study.

### Statistical Analysis

Data were analyzed using SPSS version 20. Results of descriptive analysis were tabulated in the form of median, mean and standard deviation for each individual item, as well as for total score and for the three sub-scales. Differences between groups were tested using the Kruskall-Wallis test.

## RESULTS

Out of 400 students, 195 completed and returned the questionnaires. The overall response rate was 48.8%. The response rate at KSU was 20.6% and 81.3% for males and females respectively. At Faculty of Medicine, PNU the response rate was 66.7%, Table-I.

**Table-I:** Response rate of Completed] Questionnaires according to College and Gender.

Variable	Official enrollees		Participants	
	N	(%)	N	%
King Saud University	328		149	
Male	194	59.1	40	20.6
Female	134	40.9	109	81.3
Princess Nora University(F)	72		46	63.9
Total	400		195	48.8

Mean scores of individual items are shown in Table-II, Out of the 13 items measuring self-management, only three scored 4 and above, the other items showed very close values. It is notable that most items of the desire for learning scored above 4, similar results were recorded for the self-control scale.

**Table-II:** Mean Scores of Individual Items of the SDLRS.

Item	Mean	SD
*Self-Management*		
1.I solve problems using a plan	3.89	0.9
2.I prioritize my work	4.09	0.8
3.I do not manage my time well	2.95	1.1
4.I have good management skills	3.68	0.9
5.I set strict time frames	3.47	0.9
6.I prefer to plan my own learning	4.14	0.8
7.I am systematic in my learning	3.97	0.9
8.I am confident in my ability to search out information	4.0	0.9
9.I set specific times for my study	3.83	0.9
10.I am self-disciplined	3.68	1.0
11.I am disorganized	2.68	1.2
12.I am methodical	3.75	0.8
13.I can be trusted to pursue my own learning	4.01	0.9
*Desire for Learning*		
1.I need to know why	4.33	0.8
2.I critically evaluate new ideas	4.13	0.8
3.I learn from my mistakes	4.25	0.8
4.I am open to new ideas	4.29	0.7
5.When presented with a problem I cannot resolve,I will ask for assistance	3.77	1.1
6.I like to evaluate what I do	4.10	0.9
7.I do not enjoy studying	2.80	1.2
8.I have a need to learn	4.17	0.9
9.I enjoy a challenge	4.21	0.9
10.I want to learn new information	4.39	0.8
11.I enjoy learning new information	4.38	0.7
12.I like to gather the facts before I make a decision	4.18	0.9
Self-control		
1.I am able to focus on a problem	4.22	0.8
2.I prefer to set my own learning goals	4.22	0.8
3.I am responsible	4.32	0.8
4.I have high personal expectations	4.33	0.8
5.I have high personal standards	4.34	0.7
6.I have high beliefs in my abilities	4.32	0.8
7.I am aware of my own limitations	4.06	0.8
8.I am logical	4.26	0.8
9.I evaluate my own performance	4.14	0.9
10.I prefer to set my own criteria on which to evaluate my performance	4.11	0.8
11.I am responsible for my own decisions/actions	4.46	0.7
12.I can find out information for myself	4.18	0.9
13.I like to make decisions for myself	4.23	0.9
14.I prefer to set my own goals	4.36	0.8
15.I am not in control of my life	2.60	1.2

Table-III showed the mean score for the three scales and for the total scale. It is evident that the highest score was for desire for learning (4.08±0.5) followed by self-control (3.97±0.9), while the least score was for self-management (3.7 ± 0.5). Identical results were noticed for both males and females at KSU. Results of PNU and KSU were very close for the desire for learning and self-control. As for self-management, PNU score was 3.46 compared to 3.69 for KSU. Overall, no statistical differences were found between KSU and PNU students for the three scales and for the total scale (p>0.05).

**Table-III:** Comparison of the study groups according to the values of the three scales of the SDLRS.

	Self-management			Desire for learning			Self-control		
	KSU Males	KSU Females	PNU	KSU Males	KSU Females	PNU	KSU Males	KSU Females	PNU
Mean	3.73	3.76	3.54	4.09	4.10	4.03	3.76	4.02	4.04
SD	0.6	0.5	0.4	0.6	0.5	0.4	1.3	0.9	0.5
Median	3.69	3.69	3.46	4.17	4.17	4.0	4.1	4.20	4.0
Total mean score	3.7 ± 0.5			4.08 ± 0.5			3.97 ± 0.9		

## DISCUSSION

Medical students need to acquire a number of learning skills as confidence, autonomy, motivation and preparation for lifelong learning.^10^ SDL is one of the skills that is essential for medical students to be life- long learners especially in the medical curriculum adopting PBL.^4^,^9^ Integration of SDL in a curriculum, would help deep understanding, memorizing the content, and promote the exchange of ideas. Overall results of the present study indicated that both KSU and PNU students have positive attitude toward readiness for SDL. This is in accordance with other studies from other Saudi Universities.^11^,^12^

In spite of the challenges being faced by the newly enrolled medical students in Saudi Arabia, due to language barrier, and that the program is offered as an undergraduate program yet, the present study demonstrated that students have high desire for learning indicating a positive attitude. This is an encouraging finding and is considered to be a positive outcome. Similar results were reported by earlier studies showing that medical students have high desire for learning.^13^-^15^ This finding can be explained by the unified admission criteria for most of Medical schools world-wide which recruit the top students who demonstrated excellent performance all throughout their education.^16^ Our study also demonstrated that this high desire for learning is not unique for any of the study groups where the score is identical for both males and females and also showed no statistical difference between KSU and PNU, even though the preparatory year of PNU students was not at the same level as compared to KSU.

Regarding the self-control, the mean score was approaching 4 with closely related values for all the study groups. This high result indicated great confidence and maturity of our students. In comparison to the encouraging results of the desire for learning and self-control, the self-management scale have lower values, being 3.69 for KSU and 3.46 for PNU. This result indicated that students need support in self-management skills especially in planning, time management and in utilizing systematic methodology for learning. Based on the results of the present study, it is advised that action plans should be taken to improve self-management skills of first year medical students. This can be achieved through extra circular workshops specially designed to tackle specific items.^17^ Furthermore, the low scored items could be addressed using an innovative approach aimed at behavioral changes during the learning skills and professionalism courses which are whole year courses provided for both KSU and PNU during the first two years of the medical program. It would also be of great help to arrange hands-on workshops for the students as well as planning for peer tutorials during the SDL time.

### Limitations of the study

Small sample size of male participants and of PNU respondents.

## CONCLUSION

The present study provides baseline data about readiness of first year Saudi medical students for SDL. Students demonstrated high desire for learning and self-control, yet the self-management skills needs further improvement which can be achieved through multidisciplinary approaches.
